# Released Exosomes Contribute to the Immune Modulation of Cord Blood-Derived Stem Cells

**DOI:** 10.3389/fimmu.2020.00165

**Published:** 2020-02-25

**Authors:** Wei Hu, Xiang Song, Haibo Yu, Jingyu Sun, Yong Zhao

**Affiliations:** ^1^Center for Discovery and Innovation, Hackensack Meridian Health, Nutley, NJ, United States; ^2^Department of Chemistry and Chemistry Biology, Stevens Institute of Technology, Hoboken, NJ, United States

**Keywords:** exosome, immune modulation, CB-SC, T cell, monocyte, M2 macrophage, stem cell educator therapy

## Abstract

**Background:** Clinical studies demonstrated the immune modulation of cord blood-derived stem cells (CB-SC) for the treatment of type 1 diabetes and other autoimmune diseases, with long-lasting clinical efficacy. To determine the molecular mechanisms underlying the immune modulation of CB-SC, the actions of exosomes released from CB-SC were explored in this study.

**Methods:** Exosomes were isolated from CB-SC cultures using ultracentrifugation and confirmed with different markers. The activated T cells and purified monocytes from peripheral blood mononuclear cells (PBMC) were treated with CB-SC in the presence or absence of the purified exosomes, followed by functional and flow cytometry analysis of phenotypic changes with different immune cell markers.

**Results:** CB-SC-derived exosomes displayed the exosome-specific markers including CD9, CD63, and Alix, at the size of 85.95 ± 22.57 nm. In comparison with the treatment of CB-SC, functional analysis demonstrated that the CB-SC-derived exosomes inhibited the proliferation of activated PBMC, reduced the production of inflammatory cytokines, downregulated the percentage of activated CD4^+^ T and CD8^+^ T cells, and increased the percentage of naive CD4^+^ T and CD8^+^ T cells. Using the fluorescence dye DiO-labeled exosomes, flow cytometry revealed that exosomes preferably bound to the monocytes in the PBMC, leading to an improvement of mitochondrial membrane potential of treated monocytes. Further study indicated that the purified monocytes gave rise to spindle-like macrophages displaying type 2 macrophage (M2) surface markers and upregulating an expression of immune tolerance-related cytokines after the treatment with exosomes.

**Conclusions:** CB-SC-derived exosomes display multiple immune modulations and primarily on monocytes, contributing to the immune education of CB-SC in the clinical treatment of autoimmune diseases.

## Introduction

Autoimmunity, leading to the destruction of pancreatic islet β cells in type 1 diabetes (T1D), is mediated by abnormalities in multiple types of immune cells, including T cells, B cells, regulatory T cells (Treg), monocytes/macrophages (Mo/Mφ), and dendritic cells (DC) (1). While insulin therapy allows T1D patients to manage blood sugar, it does not address the underlying immune dysfunction. Pancreatic and islet transplantations can overcome the shortage of insulin-producing islet β cells. However, donor scarcity and risk of immune rejection severely limit the use of these treatment modalities. Insulin-producing cells have been generated from embryonic stem (ES) cells and induced pluripotent stem cells (iPSC) through *ex vivo* inductions (2). However, transplanting these stem cells can also cause immune rejection (3), raising ethical and safety concerns. Alternative approaches are needed to correct the underlying autoimmunity of T1D.

We developed the Stem Cell Educator (SCE) therapy, which harnesses the unique therapeutic potential of cord blood-derived stem cells (CB-SC) to treat the multiple immune dysfunctions in diabetes (4–7). SCE therapy circulates patient's blood through a blood cell separator, cocultures the patient's lymphocytes with adherent CB-SC *in vitro*, and returns “educated” lymphocytes to the patient's circulation (4, 5, 7). In contrast to conventional immune therapies, SCE therapy modifies rather than destroys the cells responsible for autoimmunity. Our clinical phase 1/2 trials indicate that SCE therapy reverses autoimmunity, promotes regeneration of islet β cells, and improves metabolic control for the treatment of T1D (4–7) and T2D (6, 7). SCE therapy elicits immune tolerance by altering autoimmune T cells and pathogenic Mo/Mφs through the autoimmune regulator (AIRE) and other molecular pathways (8). Exosomes are the smallest extracellular vesicles (EVs) (30–150 nm) that are produced by a variety of cells, and existing in all biological fluids, with diverse biological functions in the maintenance of homeostasis (9–11) To understand the molecular mechanisms underlying the immune education of SCE therapy to improve its clinical efficacy for the treatment of T1D and other autoimmune diseases, we explored the action of CB-SC-derived exosomes in this study. The data demonstrated multiple immune modulations of CB-SC-derived exosomes on immune cells.

## Materials and Methods

### Cell Culture for CB-SC

The culture of CB-SC was performed as previously described (4). In brief, human umbilical cord blood units (50–100 ml/U) were collected from healthy donors and purchased from Cryo-Cell International blood bank (Oldsmar, FL). Cryo-Cell has received all accreditations for cord blood collections and distributions, with hospital institutional review board (IRB) approval and signed consent forms from donors. Mononuclear cells were isolated with Ficoll-Hypaque (γ = 1.077, GE Health), and red blood cells were removed using ammonium–chloride–potassium (ACK) lysis buffer (Lonza). The remaining mononuclear cells were washed three times with phosphate-buffered saline (PBS) and seeded in 150 × 15 mm style non-tissue culture-treated Petri dishes (Becton Dickinson Labware) at 1 × 10^6^ cells/ml. Cells were cultured in X-Vivo 15 chemical-defined serum-free culture medium (Lonza) and incubated at 37°C with 8% CO_2_ for 10–14 days. To characterize the phenotype and purity of CB-SC, the detached CB-SC were performed by flow cytometry with associated markers, including leukocyte common antigen CD45, ES cell markers OCT3/4 and SOX2, hematopoietic stem cell marker CD34, and the immune tolerance-related markers CD270 and CD274. Isotype-matched immunoglobulin G (IgGs) served as control.

### Isolation of Exosomes From CB-SC Culture

At 80–90% of confluence, CB-SC were washed with PBS three times to remove all cellular debris, supplied with 25 ml fresh serum-free medium, and continued culturing. After 4 days, the conditioned medium was harvested for purifying exosomes by ultracentrifugation in an Optima L-100XP ultracentrifuge (Beckman Coulter) as previously described (12, 13). Initially, the conditioned medium was centrifuged at 2,000 g for 20 min to remove cellular debris and other components, and followed by centrifugation at 10,000 g for 30 min to remove mitochondria. Consequently, the supernatant was collected and transferred to a new tube with a 10-kDa Amicon® Ultra-15 Centrifugal Filter Unit (Millipore Sigma) and centrifuged at 4,000 g for 30 min to concentrate the supernatant, followed by centrifugation at 100,000 g for 70 min. Finally, the pellets were rewashed once with PBS at 100,000 g for 70 min. The purified exosomes were resuspended in 100 μl PBS and transferred to a new centrifuge tube with a 0.22-μm filter and centrifuged at 2,000 g for 2 min. All centrifugation was performed at 4°C. The purified exosomes were kept at −80°C for later applications.

## Characterization OF Exosome

### Electron Microscopy

The concentrated exosomes were loaded onto the electron microscope grids coated with Formvar. After being contrasted with uranyl–acetate solution and embedded in methylcellulose, exosomes were observed and photographed with a FEI Titan Themis 200 kV scanning transmission electron microscope (Thermo Fisher Scientific).

### Dynamic Light Scattering

The size of the exosomes was determined using a dynamic light scattering (DLS) method, performed with a Nano-ZS Zetasizer Analyzer (Malvern Instruments Ltd, Malvern, united Kingdom) with a refractive index (RI) at 1.39.

### Western Blotting

Purified exosomes or cells were treated with radioimmunoprecipitation assay (RIPA) buffer; protein concentration was determined by a bicinchoninic acid (BCA) protein assay. Proteins were separated by 10% Tris–HCl gel (Bio-Rad) and transferred to the polyvinylidene fluoride (PVDF) membrane, blotted overnight with anti-human Calnexin (Biolegend) and anti-human Alix monoclonal antibodies (mAb) (Biolegend), followed by anti-rat or anti-mouse horseradish peroxidase (HRP)-conjugated secondary mAb (Thermo Fisher Scientific). Membranes were incubated with chemiluminescent substrate (Millipore Sigma), and chemiluminescent signal was detected upon exposure to autoradiographic films.

### FACS Assessment of Exosomes Characterization

To further confirm the release of exosomes from CB-SC, we performed the flow cytometry analysis after being captured with Exosome-Human CD63 Isolation/Detection Beads (Thermo Fisher, Waltham, MA). Owing to the small size of CB-SC-derived exosomes and the limitation (>200 nm) of particle size detected by the Gallios Flow Cytometer (Beckman Coulter), the human CD63 isolation/detection beads (4.5-μm size) were utilized to isolate the exosomes from the ultracentrifuge-concentrated supernatant of CB-SC cultures for flow cytometry. Exosomes (10–20 μg protein) were incubated with 20 μl of 4.5-μm size anti-human CD63 beads in 100 μl volume and incubated overnight at 4°C under 500 rpm agitation. Consequently, exosomes bound with CD63-capturing beads were place in the DynaMag™-2 Magnet Stand (Thermo Fisher) and washed twice with PBS, and followed by the preparation for flow cytometry. Exosomes captured by anti-CD63 beads were aliquoted and incubated with phycoerythrin (PE)-conjugated anti-human CD63 (BD Bioscience), fluorescein isothiocyanate (FITC)-conjugated anti-human CD81 (BD Bioscience), and PE-conjugated anti-human CD9 mAb (BD Bioscience), respectively, for 45 min at room temperature and then washed twice with PBS in the DynaMag™-2 Magnet Stand, followed by flow cytometry.

### PBMC Isolation and Proliferation Assay

Human buffy coat blood units were purchased from the New York Blood Center (New York, NY). Human peripheral blood-derived mononuclear cells (PBMC) were harvested as previously described (5). PBMC were stained with carboxyfluorescein succinimidyl ester (CFSE) (Life Technologies) according to the manufacturer protocol, then stimulated with Dynabeads coupled with anti-CD3 and anti-CD28 antibodies (Life Technologies) for 72 h in the presence of treatment with exosomes at 10 μg/ml, 20 μg/ml, and 40 μg/ml in duplicate, respectively, and were incubated at 37°C in 5% CO_2_ in the tissue culture-treated 96-well plate. Exosome-untreated cells served as control.

### Flow Cytometry

Flow cytometric analyses of surface and intracellular markers were performed as previously described (6). Samples were preincubated with human BD Fc Block (BD Pharmingen) for 15 min at room temperature and then directly aliquoted for different antibody staining. Cells were incubated with different mouse anti-human mAb from Beckman Coulter (Brea, CA), including PE-conjugated anti-CD56 and anti-CCR7; PE-Cy5-conjugated anti-CD19; PE-Cy7-conjugated anti-CD11c, and anti-CD45, anti-CD45RO; allophycocyanin (APC)-conjugated anti-CD80; APC-Alexa Fluor 750-conjugated anti-CD8 and anti-CD86; Krome Orange-conjugated anti-CD14; and PC 5.5-conjugated human leukocyte antigen DR isotype (HLA-DR). From BD Biosciences (San Jose, CA), mAb include the following: the APC-conjugated anti-human CD4 antibody, PE-conjugated anti-CD63 and anti-CD163, FITC-conjugated anti-CD81 and anti-CD206, Alexa Fluor 488-Sox2 and BV421-conjugated anti-CD209, and propidium iodide (PI). The eFluor 660-conjugated rat anti-human OCT3/4 and isotype-matched IgG Abs and FITC-conjugated CD9 were from Thermo Fisher. Pacific Blue (PB)-conjugated anti-human CD3 Ab was from BioLegend. For surface staining, cells were stained for 30 min at room temperature and then washed with PBS at 2,000 rpm for 5 min before flow analysis. Isotype-matched mouse anti-human IgG antibodies (Beckman Coulter) served as a negative control for all fluorescein-conjugated IgG mAb. For intracellular staining, cells were fixed and permeabilized according to the PerFix-nc kit (Beckman Coulter) manufacturer's recommended protocol. After staining, cells were collected and analyzed using a Gallios Flow Cytometer (Beckman Coulter) equipped with three lasers (488 nm blue, 638 nm red, and 405 nm violet lasers) for the concurrent reading of up to 10 colors. The final data were analyzed using the Kaluza Flow Cytometry Analysis Software version 2.1 (Beckman Coulter).

### BCA Assay

Exosome concentration was measured by protein quantification using BCA assay (14), using the Pierce™ BCA Protein Assay Kit (Thermo Fisher Scientific). For protein concentration quantification, 10 μl of exosome samples were incubated with 200 μl BCA reagent (Thermo Fisher Scientific) at 37°C for 30 min. Absorbance was read at 562 nm within 10 min. The protein concentration was determined by interpolating test sample concentrations relative to the standard concentration curve using bovine serum albumin.

### Assessment of Exosome Uptake by Different Subpopulation PBMC

To explore the interaction of CB-SC-derived exosomes with different cell compartments of PBMC, PBMC were incubated in non-tissue-treated hydrophobic 24-well plates (avoiding the attachment of monocytes), with the green fluorescent lipophilic dye 3,3′-dioctadecyloxacarbocyanine perchlorate (DiO) (Millipore Sigma)-stained exosomes. Four hours later, different cell populations in PBMC were labeled with mAb specific for lineage markers including Pacific Blue-conjugated anti-human CD3 (Thermo Fisher Scientific), APC-conjugated anti-human CD4 (BD Bioscience), AF750-conjugated anti-human CD8 (Beckman Coulter), PE-Cy7-conjugated anti-human CD11c (Beckman Coulter), Krome Orange-conjugated anti-human CD14 (Beckman Coulter), PC5-conjugated anti-human CD19 (Beckman Coulter), and PE-conjugated anti-human CD56 mAb (Beckman Coulter). To determine T-cell population and remove CD4^+^ monocytes, anti-CD3 Ab was employed for gating out CD4^+^ monocytes, in addition to the consideration of cell-size difference.

### Action of CB-SC Derived Exosomes on Monocytes

Monocytes were purified from PBMC using CD14^+^ microbeads (Miltenyi Biotec) according to the manufacturer's instruction, with purity of CD14^+^ cells >95%. The purified CD14^+^ monocytes were initially seeded in the tissue culture-treated six-well plate at 5 × 10^5^ cells/well and cultured in X-Vivo 15 serum-free medium, at 37°C, 5% CO_2_ conditions. After adhering for 2 h, the attached monocytes were washed twice with PBS to remove all floating cells and cell debris, followed by treatment with or without CB-SC-derived exosomes (40 μg/ml) in X-Vivo 15 serum-free medium, at 37°C, 5% CO_2_ conditions. After treatment for 3 days, both the supernatant and detached cells were collected for ELISA and flow cytometry, respectively. The morphological change was photographed by phase-contrast microscope before cells were detached with 0.25% trypsin/ethylenediaminetetraacetic acid (EDTA) (Corning, New York) or an 18-cm cell scraper (BD Falcon). The exosome-treated and untreated cells were immunostained with a combination of mAbs, including Krome Orange-conjugated anti-human CD14 (Beckman Coulter), APC-conjugated anti-human CD80 (Beckman Coulter), AF750-conjugated anti-human CD86 (Beckman Coulter), PE-conjugated anti-human CD163, FITC-conjugated anti-human CD206, and BV421-conjugated anti-human CD209 (BD Bioscience) for 30 min at room temperature and then washed with PBS at 2,000 rpm for 5 min before flow analysis. Isotype-matched mouse anti-human IgG antibodies (Beckman Coulter) served as a negative control.

To examine the effects of CB-SC-derived exosomes on mitochondrial function of monocytes, 3 × 10^5^ fresh PBMC were plated on non-tissue-treated hydrophobic 24-well plates (avoiding the attachment of monocytes); PBMC were cultured with or without CB-SC-derived exosomes. After incubation for 3 h, the PBMC were harvested to be stained with anti-human CD14-Krome Orange-conjugated antibody and cytoplasmic Ca^2+^ dye (Fluo-4) (Thermo Fisher Scientific) and mitochondrial Ca^2+^ dye (Rhod-2) (Thermo Fisher Scientific) and tetramethylrhodamine, ethyl ester (TMRE) (Abcam) for the detection of mitochondrial membrane potential.

### ELISA Assay

To detect the cytokine production by PBMC, 1 × 10^5^ PBMC were stimulated with anti-CD3/anti-CD28 beads in the presence or absence of CB-SC-derived exosomes at 10, 20, and 40 μg/ml in triplicate in a 96-well plate with a total of 200 μl X-Vivo 15 serum-free culture medium (Lonza) per well. After the treatment for 72 h, the supernatants were collected to examine the inflammatory cytokines tumor necrosis factor alpha (TNF-α) and interferon gamma (IFN-γ) using human TNF-α and IFN-γ enzyme-linked immunosorbent assay (ELISA) kits (Biolegend) according to the manufacturer protocols, respectively.

To examine the cytokine production by monocytes, the purified CD14^+^ monocytes were initially seeded in the tissue culture-treated six-well plate at 5 × 10^5^ cells/well and cultured in X-Vivo 15 serum-free medium, at 37°C, 5% CO_2_ conditions. After adhering for 2 h, the attached monocytes were washed twice with PBS to remove all floating cells and cell debris, followed by treatment with or without CB-SC-derived exosomes (40 μg/ml) in X-Vivo 15 serum-free medium, at 37°C, 5% CO_2_ conditions. After treatment for 3 days, supernatants were collected to test the transforming growth factor (TGF)-β1 and interleukin (IL)-10 using ELISA kits (Biolegend) according to manufacturer's recommended protocol, respectively.

### Statistical Analysis

Statistical analyses were performed with GraphPad Prism 8 (version 8.0.1) software. The normality test of samples was performed by the Shapiro–Wilk test. Statistical analyses of data were performed by the two-tailed Student's *t*-test to determine statistical significance for parametric data. Mann–Whitney *U*-test was utilized for non-parametric data. Values were given as mean ± standard deviation (SD). Statistical significance was defined as *P* < 0.05, with two sided.

## Results

### Characterization of CB-SC-Derived Exosomes

**Figure 1:**
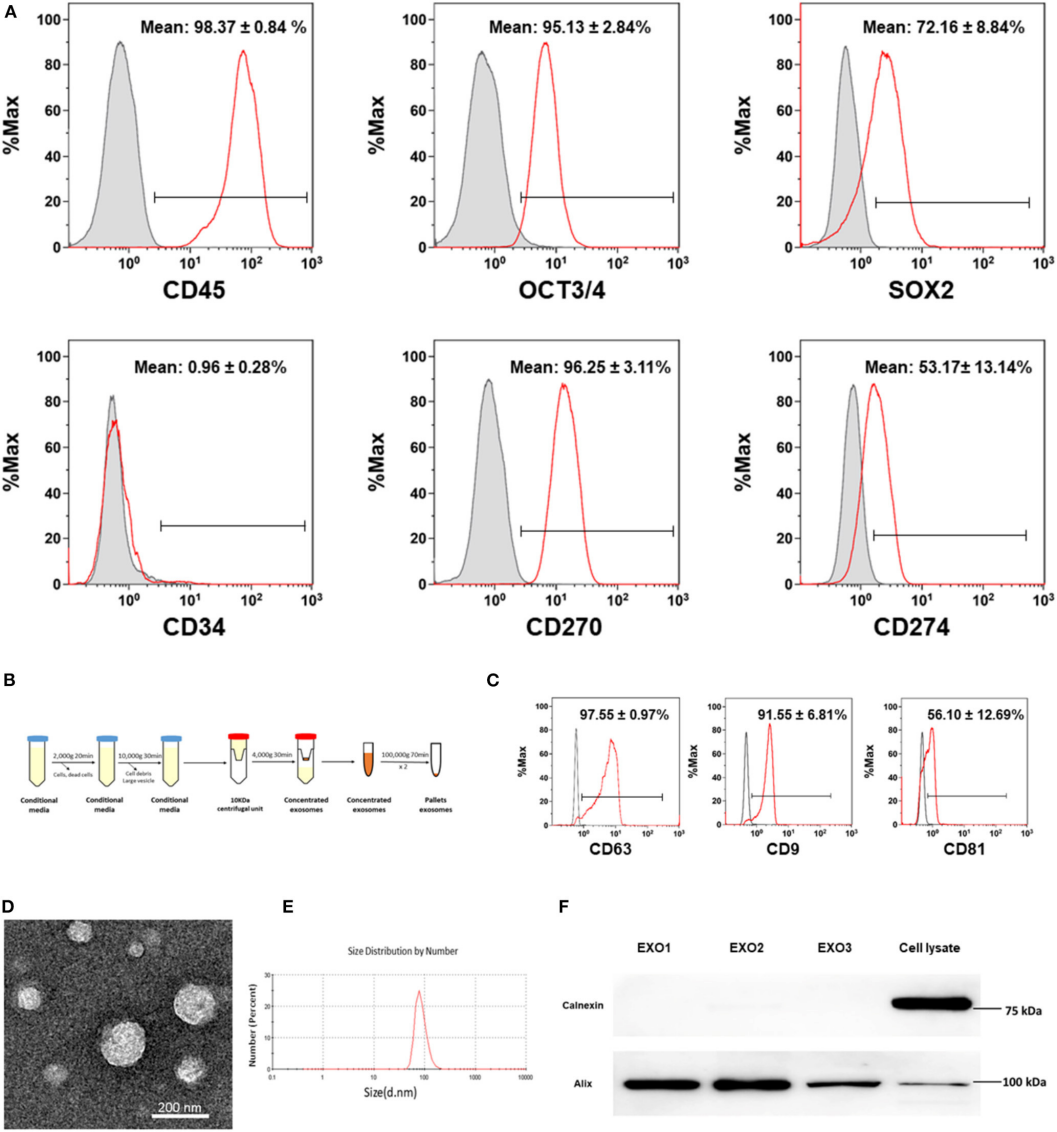
Characterization of cord blood multipotent stem cell (CB-SC)-derived exosomes. **(A)** Phenotypic characterization of CB-SC with a high purity. The detached CB-SC were performed by flow cytometry with associated markers including leukocyte common antigen CD45, embryonic stem (ES) cell markers OCT3/4 and SOX2, hematopoietic stem cell marker CD34, and the immune tolerance-related markers CD270 and CD274. Isotype-matched immunoglobulin G (IgGs) served as control. Data were represented from three experiments with similar results. **(B)** Outline the protocol for an isolation of CB-SC-derived exosomes. **(C)** Flow cytometry analysis of the expression of CB-SC-derived exosome-specific markers CD63, CD9, and CD81. The CB-SC-derived exosomes (*N* = 4) were initially concentrated by ultracentrifugation and followed by capturing with anti-CD63 Dynabeads. Isotype-matched IgGs served as control (gray histogram). **(D)** Image of CB-SC-derived exosomes by electron microscopy. **(E)** Size distribution of CB-SC-derived exosomes. **(F)** Western blotting of CB-SC-derived exosomes with exosome marker Alix and ER-associated marker calnexin.

Initially, the phenotype and purity of CB-SC were characterized by flow cytometry with CB-SC-associated markers (8, 15) including leukocyte common antigen CD45, ES cell markers OCT3/4 and SOX2, hematopoietic stem cell marker CD34, and the immune tolerance-related markers CD270 and CD274. CB-SC highly express CD45, OCT3/4, SOX2, and CD270, with medium level of CD274 and no expression of CD34 (Figure 1A). CD45 and OCT3/4 are regularly utilized for the purity test of CB-SC, at ≥95% of CD45^+^OCT3/4^+^ CB-SC.

Next, exosomes were purified from CB-SC cultures using serial centrifugations (Figure 1B). Phenotypic characterization confirmed the expression of exosome-specific markers such as CD9 and CD81 on the CB-SC-derived exosomes by flow cytometry, which were analyzed following the purification by conjugation with anti-CD63 beads (Figure 1C). The presence of exosomes was demonstrated by transmission electron microscopy (Figure 1D), with the size of 85.95 ± 22.57 nm (Figure 1E). Western blot further proved the expression of the exosome-associated universal marker Alix, but failed to exhibit the endoplasmic reticulum (ER)-associated marker calnexin (Figure 1F). The data indicated that CB-SC release exosomes.

### Suppression of PBMC Proliferation by CB-SC-Derived Exosomes

**Figure 2:**
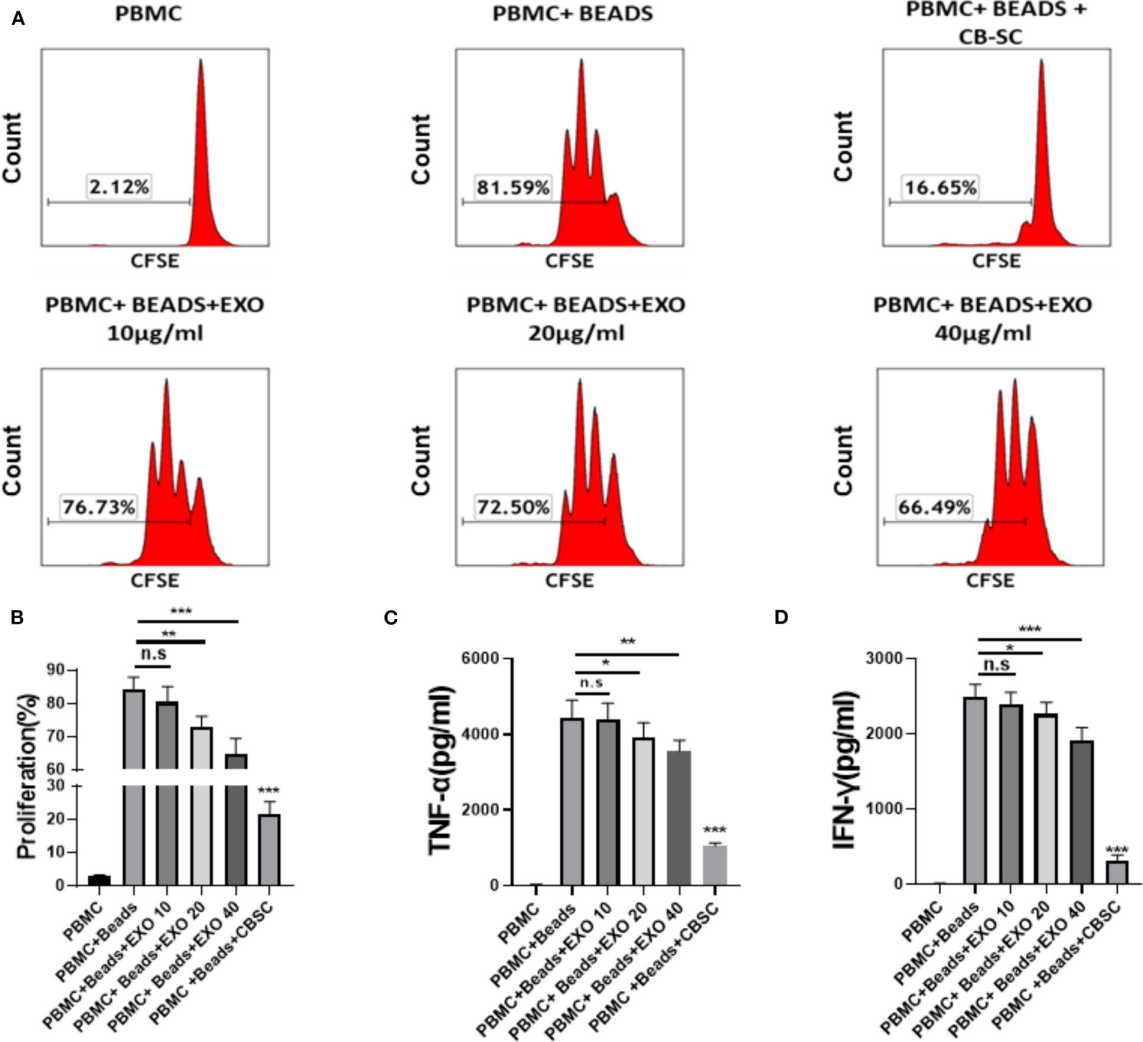
Modulation of cord blood-derived stem cell (CB-SC)-derived exosomes on peripheral blood mononuclear cell (PBMC). **(A–D)** Health donor-derived PBMC (*N* = 3) were treated with CB-SC-derived exosomes (*N* = 3) for 3 days in the presence of T-cell activator CD3/CD28 Dynabeads. **(A)** Suppression of PBMC proliferation by CB-SC-derived exosomes. The carboxyfluorescein succinimidyl ester (CFSE)-labeled PBMC were stimulated to proliferate with T-cell activator CD3/CD28 Dynabeads in the presence of different dosages of CB-SC-derived exosomes. Treatment with CB-SC served as positive control. Untreated PBMC served as negative control. Histograms of flow cytometry were representative of nine experiments with similar results. **(B)** Quantitative analysis of PBMC proliferation showed a marked reduction in PBMC expansion after the treatment with CB-SC-derived exosomes at the dosage of 10 μg/ml (*p* > 0.05), 20 μg (*p* < 0.01)/ml, and 40 μg/ml (*p* < 0.005), respectively. **(C)** Suppression of inflammatory cytokine tumor necrosis factor alpha (TNF-α) production in presence of different dosages of CB-SC-derived exosomes at 10, 20, and 40 μg/ml. **(D)** Decrease in the level of cytokine IFN-γ in PBMC after the treatment with different dosages of CB-SC-derived exosomes at 10, 20, and 40 μg/ml. Data were given as mean ± SD (standard deviation). *p < 0.05; **p < 0.01; ***p < 0.005.

To explore the immune modulation of CB-SC-derived exosomes, the anti-CD3/CD28 bead-activated PBMC were initially treated with different dosages of CB-SC-derived exosomes ranging from 10 to 40 μg/ml. The PBMC proliferation was evaluated by carboxyfluorescein succinimidyl ester (CFSE) staining and flow cytometry analysis. The data demonstrated that the proliferation of PBMC was markedly declined after the treatment with CB-SC, with a percentage reduction about of 61.55 ± 6.43% (Figures 2A,B). In comparison, treatment with different dosages of exosomes declined the percentage of PBMC proliferation at 5.54% for the dosage of 10 μg/ml exosomes, 10.99% for 20 μg/ml, and 15.37% for 40 μg/ml, respectively (Figure 2A). There were significant differences for the dose groups at 20 μg/ml (*P* < 0.01) and 40 μg/ml (*P* < 0.005) relative to the group of anti-CD3/CD28-activated PBMC (Figure 2B).

In addition, we tested the inhibitory effect of CB-SC-derived exosomes at high doses such as 80 and 160 μg/ml on the anti-CD3/CD28 bead-activated PBMC. The inhibitory effect of exosomes on the proliferation of anti-CD3/CD28 bead-activated PBMC was markedly improved at these high dosages (Figure S1). In consideration of the clinical design (4, 7), the 9- or 12-layer Stem Cell Educators are utilized for the treatment of patients. Based on the current protocol, a maximum of 80 μg exosomes was normally purified from 240 ml supernatants of CB-SC cultures (20 ml per layer for 12 layers). Therefore, the optimal dose of exosomes at 40 μg/ml was utilized for the following experiments. Meanwhile, inflammatory cytokines were examined by ELISA assay. We found that the levels of TNF-α and IFN-γ were markedly decreased following treatment with CB-SC-derived exosomes at 20 μg/ml (*P* < 0.05) and 40 μg/ml (*P* < 0.01 and *p* < 0.005 respectively) (Figures 2C,D). In addition, the CD4/CD8 ratio was analyzed, and it failed to display a marked change after the treatment with exosomes, with CD4/CD8 ratios at 2.49 ± 0.97 for the PBMC group, 2.53 ± 0.94 for the PBMC + beads group, and 2.49 ± 0.96 for the PBMC + beads + exosomes group (*P* > 0.05, *N* = 5).

### Downregulation of Activated T Cells and Increasing Naive T Cells by CB-SC-Derived Exosomes

**Figure 3:**
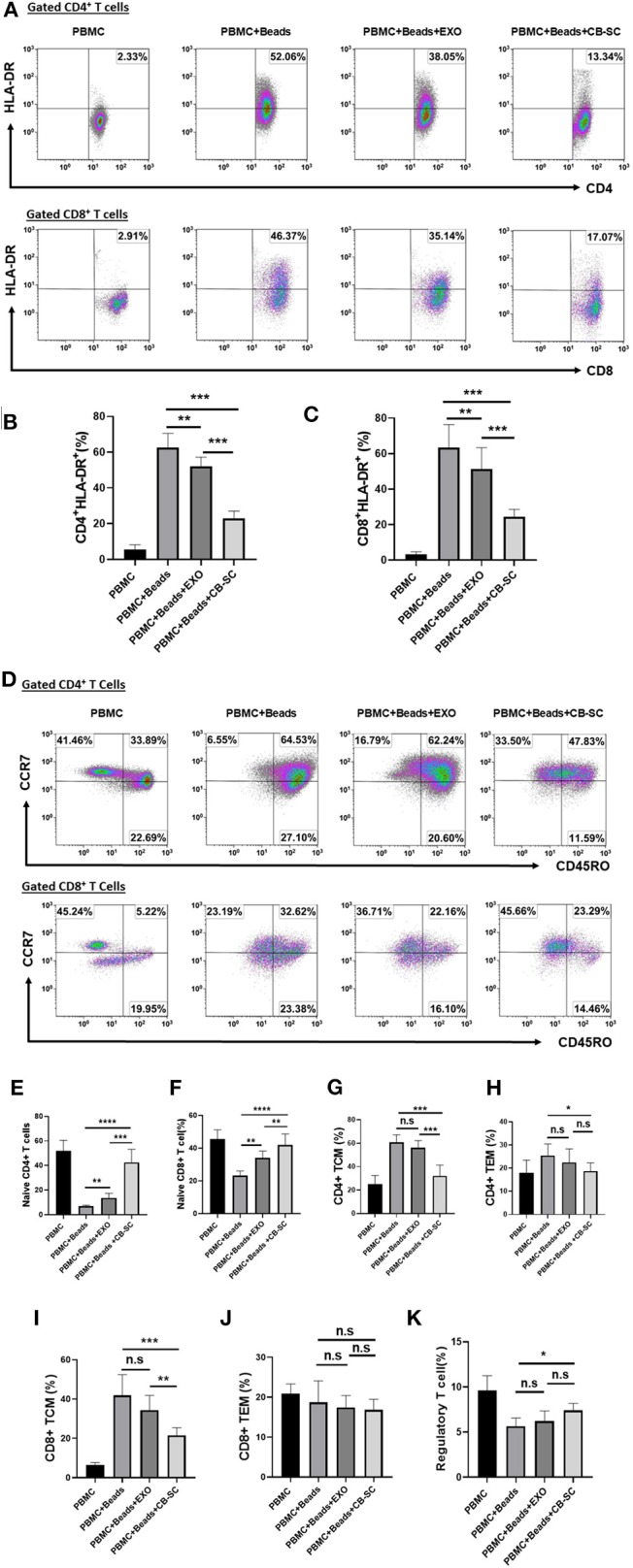
Modulation of different T-cell subpopulations by cord blood-derived stem cell (CB-SC)-derived exosomes. **(A–J)** Peripheral blood mononuclear cells (PBMC) (*N* = 3) were activated with T-cell activator CD3/CD28 Dynabeads in the presence of 40 μg/ml exosomes derived from CB-SC cultures (*N* = 8). Treatment with CB-SC served as positive control. Untreated PBMC served as negative control. **(A)** Downregulation of activated CD4^+^ and CD8^+^ T cells by CB-SC-derived exosomes. Histograms of flow cytometry were representative of five experiments with similar results. **(B)** Decline the level of human leukocyte antigen DR isotype (HLA-DR) expression on the activated CD4^+^ T cells by CB-SC-derived exosomes. **(C)** Decline the level of HLA-DR expression on the activated CD8^+^ T cells by CB-SC-derived exosomes. **(D)** Upregulation of the percentage of naive CD4^+^ and CD8^+^ T cells by CB-SC-derived exosomes. Histograms of flow cytometry were representative of five experiments with similar results. **(E)** Increase in the percentage of naive CD4^+^ T cells by CB-SC-derived exosomes. **(F)** Increase in the percentage of naive CD8^+^ T cells by CB-SC-derived exosomes. **(G–K)** There were no significant effects on the percentages of CD4^+^ T_CM_ cells **(G)**, CD4^+^ T_EM_ cells **(H)**, CD8^+^ T_CM_ cells **(I)**, CD8^+^ T_EM_ cells **(J)**, and CD4^+^CD25^+^CD127^dim/−^ Treg cells **(K)** after the treatment with CB-SC-derived exosomes. Results were given as mean ± SD. *p < 0.05; **p < 0.01; ***p < 0.005; ****p < 0.001.

A previous work demonstrated CB-SC modulation of activated and memory T cells after the treatment with SCE therapy in type 1 diabetic subjects (5). To explore the action of CB-SC-derived exosomes, the anti-CD3/CD28-activated PBMC were examined after the treatment with CB-SC-derived exosomes. Flow cytometry revealed that both percentages of activated CD4^+^HLA-DR^+^ and CD8^+^HLA-DR^+^ T cells were reduced about 8–14% in the presence of 40 μg/ml CB-SC-derived exosomes (Figures 3A–C). Further analysis with memory T-cell markers indicated that both percentages of naive CD4^+^ T cells (CD4^+^CD45RO^−^CCR7^+^) and naive CD8^+^ T cells (CD8^+^CD45RO^−^CCR7^+^) were increased posttreatment with CB-SC-derived exosomes (Figures 3D–F), but failed to show significant changes on the percentages of CD4^+^ central memory (CD4^+^ T_CM_, Figure 3G), CD4^+^ effector memory T_EM_ (CD4^+^ T_EM_, Figure 3H) cells, CD8^+^ T_CM_ (Figure 3I), CD8^+^ T_EM_ (Figure 3J), and CD4^+^CD25^+^CD127^dim/−^ Tregs (Figure 3K). In contrast, treatment with CB-SC displayed the significant downregulation of percentages of CD4^+^ T_CM_, CD4^+^ T_EM_, and CD8^+^ T_CM_ (Figures 3G–I), and upregulation of the percentage of CD4^+^CD25^+^CD127^dim/−^ Treg (Figure 3K).

### CB-SC-Derived Exosomes Primarily Target Monocytes in PBMC

**Figure 4:**
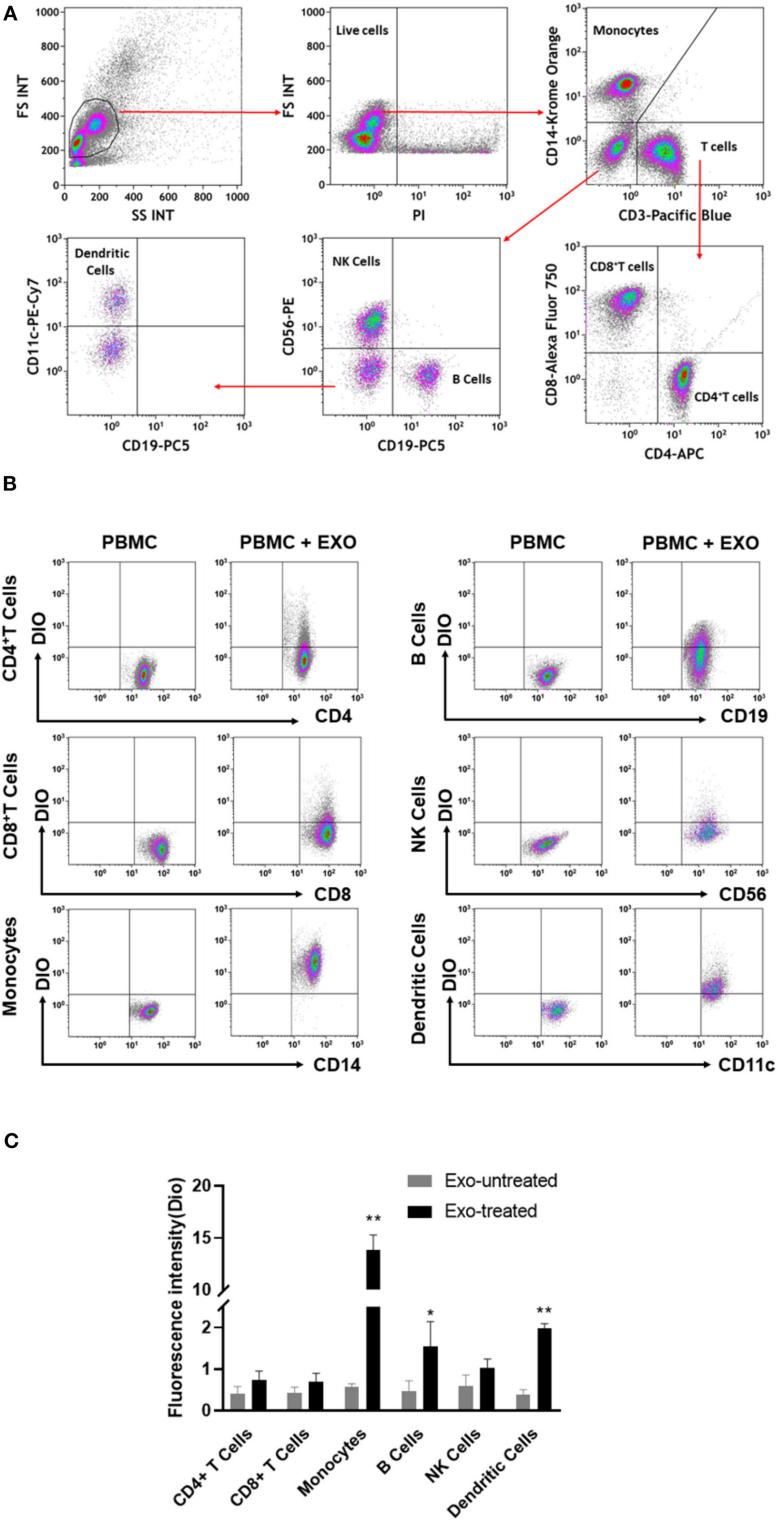
Interaction of cord blood-derived stem cell (CB-SC)-derived exosomes with different compartments of immune cells. **(A)** Flow cytometry analysis and the gating strategy with the lineage-specific surface markers for different cell compartments in PBMC (*N* = 3), including CD3/CD4/CD8 for T cells, CD19 for B cells, CD14 for monocytes, CD11c for dendritic cells, and CD56 for NK cells. **(B)** Flow cytometry revealed the distributions of DiO-labeled CB-SC-derived exosomes (*N* = 3) among different cell populations at different levels. **(C)** CB-SC-derived exosomes (*N* = 3) primarily targeted on monocytes with high fluorescence intensity. Data were representative of three experiments with six preparations of CB-SC-derived exosomes.

To further explore the action of CB-SC-derived exosomes on other immune cells, PBMC were treated with CB-SC-derived exosomes labeled with DiO dye. Different blood lineage cells were characterized and gated with cell-specific markers such as CD3 for T cells, CD4 for CD3^+^CD4^+^ T cells, CD8 for CD3^+^CD8^+^ T cells, CD11c for myeloid dendritic cells (DC), CD14 for monocytes, CD19 for B cells, and CD56 for NK cells (Figure 4A). After an incubation for 4 h, flow cytometry demonstrated that different blood cell compartments displayed at different levels of DiO-positive exosomes (Figure 4B). Notably, monocytes exhibited higher fluorescence intensity of DiO-positive exosomes than those of other immune cells, about six times higher than that of DC (Figure 4C). It suggested that CB-SC-derived exosomes were mainly found in monocytes.

### Differentiation of Monocytes Into Type 2 Macrophage (M2) After *ex vivo* Treatment With CB-SC-Derived Exosomes

**Figure 5:**
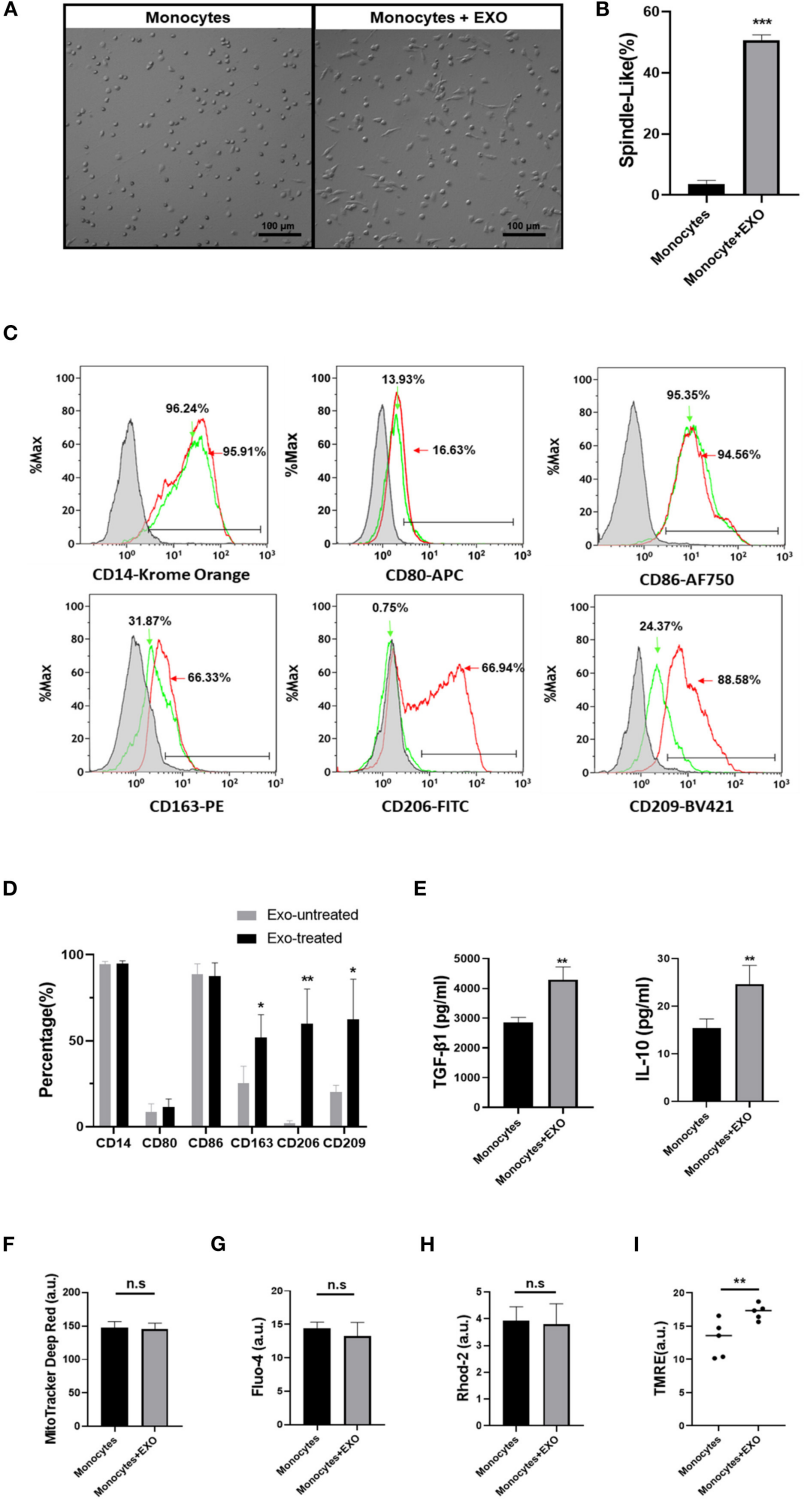
Differentiation of monocytes into type 2 macrophage (M2) after *ex vivo* treatment with cord blood-derived stem cell (CB-SC)-derived exosomes. Monocytes were purified from peripheral blood mononuclear cell (PBMC) (*N* = 3) using CD14^+^ microbeads (Miltenyi Biotec), with purity of CD14^+^ cells >95%. **(A)** Morphology change of monocytes into the spindle-like cells after the treatment with CB-SC-derived exosomes (*N* = 3). Untreated monocytes served as control (left). **(B)** Quantify the percentage of spindle-like cells in the presence of treatment with CB-SC exosomes. The data were given as mean ± SD of two experiments with three donor-derived monocytes and six preparations of CB-SC-derived exosomes (*p* < 0.001). **(C)** Upregulate the levels of M2-assoaicte markers' expressions on monocytes/macrophages after the treatment with CB-SC-derived exosomes, such as CD163, CD206, and CD209 (red line). Untreated monocytes (green line) served as control. Isotype-matched immunoglobulin G (IgG) served as negative control. Data were collected from three donor-derived monocytes and three preparations of CB-SC-derived exosomes. **(D)** Modulate the levels of M2-assoaicte markers' expressions on monocytes/macrophages after the treatment with CB-SC-derived exosomes. The data were given as mean ± SD of three PBMC (*N* =3) treated with three preparations of CB-SC-derived exosomes (*N* = 3). **(E)** Increase in the levels of transforming growth factor (TGF)-ß1 and interleukin (IL)-10 after the treatment with CB-SC-derived exosomes (*N* = 5) in three donor-derived monocytes, shown by ELISA assay. **(F)** Flow cytometry failed to show the significant difference in mitochondrial mass between exosome-treated and untreated monocytes (*N* = 3, treated with five CB-SC-derived exosomes). **(G)** Flow cytometry analysis of cytoplasmic Ca^2+^ (Fluo-4) levels failed to show the significant differences between exosome-treated and untreated monocytes. **(H)** No difference in the levels of mitochondrial Ca^2+^ (Rhod-2) between the exosome-treated and untreated monocytes. **(I)** Improve the membrane potential of mitochondria in monocytes posttreatment with CB-SC-derived exosomes, as demonstrated by flow cytometry analysis after staining with tetramethylrhodamine, ethyl ester (TMRE). The data were represented from five experiments using five donor-derived monocytes in the presence or absence of the treatment with CB-SC-derived exosomes (*N* = 5) at 40 μg/ml. The monocytes were randomly assigned the treatment with single donor CB-SC-derived exosomes. The data **(F–H)** were given as mean ± SD. *p < 0.05; **p < 0.01; ***p < 0.005.

To determine the direct effects of CB-SC-derived exosomes on monocytes, the purified CD14^+^ monocytes from PBMC were treated with CB-SC-derived exosomes. Phase contrast microscopy showed that about 50.80 ± 1.70% of exosome-treated monocytes turned into spindle-like morphologies after the treatment for 3 days (Figures 5A,B), which was similar to previously characterized fibroblast-like macrophages (16). In contrast, most of the untreated monocytes were round and spread with a few elongated cells (Figure 5A). Next, we tested their phenotype through gating the viable (PI^−^) monocytes. In comparison with untreated monocytes (Figure 5C, green histogram and Figure 5D), expressions of M2-associated markers including CD163, CD206, and CD209 were markedly increased after the treatment with CB-SC-derived exosomes, specifically for the level of CD206 expression (Figure 5C, red histogram and Figure 5D). There were no substantial changes in the expression levels of CD14, CD80, and CD86 (Figures 5C,D).

In addition, the ELISA assay further indicated the increase in the production of immune tolerance-associated cytokines such as TGF-β1 and IL-10 in the supernatant of exosome-treated monocytes (Figure 5E). Thus, these data imply that monocytes gave rise to macrophages with M2 phenotype after the treatment with CB-SC-derived exosomes.

Owing to the metabolic status of mitochondria contributing to the polarization of M1 and M2 macrophages (17, 18), we examined mitochondrial mass and associated markers following the treatment with CB-SC-derived exosomes. Labeling with MitoTracker deep red for the detection of mitochondrial mass failed to show marked difference between exosome-treated and untreated groups (Figure 5F). There were no significant differences in levels of cytoplasmic Ca^2+^ (Figure 5G) and mitochondrial Ca^2+^ (Figure 5H). However, the mitochondrial membrane potential (Δψm) was considerably upregulated after the treatment with CB-SC-derived exosomes (Figure 5I). This may contribute to polarizing the differentiation of M2 macrophages after the treatment with CB-SC-derived exosomes.

## Discussion

Exosomes are becoming one of the hot topics for pharmacology and translational medicine. The current studies demonstrated the releasing of exosomes from CB-SC cultures, with specific exosome markers. *Ex vivo* data confirmed the direct modulation of CB-SC-derived exosomes on activated PBMC and monocytes. In comparison with the substantial effects of CB-SC, the data demonstrated that CB-SC-derived exosomes inhibited the proliferation of activated PBMC, reduced the production of inflammatory cytokines, and could be effective on specific subsets of T cells such as in downregulating the percentage of activated CD4^+^ and CD8^+^ T cells and increasing the percentage of naive CD4^+^ and CD8^+^ T cells. Notably, the differentiation of purified monocytes into M2 macrophages following the treatment with CB-SC-derived exosomes provide additional molecular mechanisms underlying the immune education and induction of immune tolerance observed in the clinical use of SCE therapy for the treatment of T1D (4–6) and other autoimmune diseases like alopecia areata (19).

Previous studies have demonstrated the long-lasting clinical efficacy of SCE therapy for the treatment of T1D and T2D patients, with a complete recovery of islet β-cell functions in some subjects after a single treatment with SCE therapy sustained for 4 years (6), as well as in the treatment of alopecia areata (19). These observations suggest that SCE therapy has the potential to fundamentally correct the immune dysfunction of these subjects with autoimmune diseases. Several possible molecular mechanisms underlying the immune education of SCE therapy were reported elsewhere (8), such as an expression of autoimmune regulator (AIRE) transcription factor in CB-SC, displaying high levels of the programmed death-ligand 1 (PD-L1) and CD270 on the cell surface of CB-SC, and the release of TGF-β1 and nitric oxide (NO) (20). Current data confirmed that the released CB-SC-derived exosomes contributed to the immune down-modulation of activated CD4 and CD8 T cells, along with a corresponding increase in naive CD4 and CD8 T cells. In the process of clinical treatment, SCE therapy circulates a patient's blood through a blood cell separator, cocultures the patient's T cells and monocytes with adherent CB-SC *in vitro*, and returns “educated” autologous cells to the patient's circulation (4, 7). During the cocultures, CB-SC may release exosomes that target dysfunctional monocytes and/or T cells, leading to the tolerization of these cells and expanding the therapeutic potential of SCE therapy after infusing the cells back to the subjects. CB-SC-derived exosomes would also be transferred back to the subjects along with the CB-SC-treated patient cells, leading to the expansion of the therapeutic potential of SCE therapy in patients. Owing to their small size, the circulating CB-SC-derived exosomes may penetrate into the damaged tissues such as pancreatic islets and target the pathogenic T cells and/or macrophages, contributing to the induction of tolerance and the clearance of residential autoimmune memory T cells (21). In this respect, previous clinical study established the modulation of autoimmune T-cell memory (e.g., CD4^+^ T_CM_ and CD8^+^ T_EM_) by SCE therapy in T1D subjects (5). Current *ex vivo* study demonstrated the marked downregulation of percentages of CD4^+^ T_CM_, CD4^+^ T_EM_, and CD8^+^ T_CM_ after the treatment with CB-SC, but only slight reductions in the presence of CB-SC-derived exosomes. To overcome this limitation and improve the clinical efficacy of SCE therapy, it is necessary to further explore by which additional CB-SC-derived signals (8) contribute to the synergistic effect with exosomes in the modulation of autoimmune memory T cells. To mimic the clinical setting, it will be better to utilize T1D patient-derived PBMC, instead of using the anti-CD3/CD28-activated PBMC.

Monocytes/macrophages (Mo/Mφ) are frontline immune cells defending against viral and bacterial infections and maintaining homeostasis, with diverse functions and heterogeneity. Based on their profiles, macrophages are simply characterized with two subpopulations: type 1 macrophages (M1, proinflammation) and type 2 macrophages (M2, anti-inflammation) (22). Increasing clinical evidence and animal studies demonstrate the dysfunction of Mo/Mφ causing the pathogenesis of diabetes and other autoimmune diseases (17, 23–26). Notably, this study established that purified monocytes gave rise to cells with an M2 phenotype after the treatment with CB-SC-derived exosomes, displaying the elongated morphology and the expression of M2-associated markers (e.g., CD163, CD206, and CD209). However, the level of costimulating molecules CD80 and CD86 expressions failed to show the marked changes after the treatment with CB-SC-derived exosomes. This may be associated with our current protocol in which the purified CD14^+^ monocytes were treated with CB-SC-derived exosomes in the presence of serum-free culture medium X-Vivo 15, without adding any other growth factors such as granulocyte-macrophage colony-stimulating factor (GM-CSF) or macrophage colony-stimulating factor (M-CSF). This was different from the conventional protocol that utilized cytokines such as M-CSF, IL-4, and/or IL-10 during the M2 differentiation (27). Both CD80 (28) and CD86 expressions are normally upregulated on the activated macrophages and served as a marker for M1 (28, 29). Owing to the plasticity of monocytes/macrophages, the phenotypes of M1/M2 were significantly affected by *ex vivo* culture conditions such as the serum-containing or serum-free culture media and cytokine stimulations (27). For instance, the expression of CD80 was substantially increased on the M1 in the presence of 10% fetal bovine serum (FBS) Roswell Park Memorial Institute (RPMI) compared to the serum-free X-Vivo 10 media, but without significant difference in the level of CD86 expression between M1 and M2 (27). Therefore, culture conditions need to be optimized and standardized for the M1/M2 study.

To elucidate the mechanisms by which signaling pathway contributed to the M2 differentiation, flow cytometry revealed that the mitochondrial membrane potential (Δψm) of monocytes was markedly increased after the treatment with CB-SC-derived exosomes, without affecting both intracellular and mitochondrial calcium concentrations. Since the differentiation to a M2 macrophage is strongly favored by an increase in oxidative phosphorylation (17), the upregulation of mitochondrial membrane potential by CB-SC-derived exosomes may thus lead to the differentiation to M2 macrophages. From this perspective, Calabria and colleagues reported that the mitochondrial membrane potential of neuronal cells was recovered by the treatment with exosomes isolated from adipose stem cells (30). The reason causing the upregulation of Δψm by the interaction between exosomes and mitochondria need to be clarified in future studies. It is important to note that the differentiation of CB-SC-derived exosome-treated monocytes may be affected by patient's internal environment after being infused back during the SCE therapy. To this end, previous clinical data demonstrated that the percentage of CD86^+^CD14^+^ of monocytes was markedly reduced 4 weeks after the treatment with SCE therapy in type 2 diabetic subjects (7), even no change in the level of CD86 expression after *in vitro* treatment.

Based on the above-mentioned immune modulation potentials of CB-SC-derived exosomes, quantification of released exosomes from CB-SC cultures may provide a valuable biomarker for the Quality Control (QC) analysis of good manufacturing practice (GMP)-manufactured Stem Cell Educators before their clinical applications. However, the size of CB-SC-derived exosomes was too small at ~100 nm, which cannot be directly detected by flow cytometry and visualized by optical microscopes. It will be challenging to perform the absolute quantification with current limited technologies. The conventional approach for the isolation of exosomes was achieved by ultracentrifugation, ultrafiltration, and/or precipitation, with a duration of 4–5 h. These isolated exosomes can be quantified using current commercial techniques such as asymmetrical-flow field flow fractionation (AF4) coupled with multidetection, nanoparticle tracking analysis (NTA), DLS, and surface plasmon resonance (SPR) (31). However, due to their sensitivity, specificity, cost, and time-consuming process, the isolation and quantification of exosomes would need to be standardized with the optimized protocol. It should be noted that we utilized the conventional approach for the isolation of CB-SC-derived exosomes in the current work that might have other vesicles in different proportions. In addition, different cord blood donor-derived CB-SC may release exosomes with variable contents of bioactive molecules, and *ex vivo* functional analysis will be necessary to parallel the exosome quantification for the QC analysis of SCE products.

Previous works demonstrated that CB-SC could give rise to three germ-layer-derived cells in the presence of different inducers (15, 20), and differentiate into functional insulin-producing cells after transplantation into the chemical streptozotocin (STZ)-induced diabetic mice, leading to the reduction in hyperglycemia (15). In addition, CB-SC expressed the ES cell markers such as the self-renewal-associated transcription factors OCT3/4 and NANOG (15). Exosomes released from CB-SC might carry the capability of tissue regeneration of parent cells in addition to the immune modulation capacity. To further optimize the clinical treatment protocol and improve the clinical efficacy of SCE therapy, detailed mechanisms need to be further investigated through *ex vivo* studies such as docking of exosomes on monocytes, trafficking of exosomes in the cytoplasm of monocytes, and polarizing the M2 differentiation by the exosome treatment, along with *in vivo* studies in the autoimmune-caused diabetic mouse models (32, 33). Specifically, the proteomic analysis through mass spectrometry-based proteome profiling and exosome RNA sequencing will be necessary to dissect the detailed molecular mechanisms underlying the long-lasting clinical efficacy of SCE therapy for the treatment of T1D and other autoimmune diseases.

The current *ex vivo* study provides a better understanding of how SCE therapy results in the anti-inflammatory clinical effects. CB-SC-derived exosomes preferably and quickly bounded to monocytes in 2–3 h. As mentioned above, during the coculture of CB-SC with patient's immune cells for clinical treatment during 8–9 h (4, 5, 7, 19), the SCE-treated monocytes may carry the CB-SC-derived exosomes back into the body, probably resulting in further M2 differentiation and induction of tolerance. Therefore, SCE therapy has the potential to revolutionize the treatment of diabetes and multiple autoimmune diseases through CB-SC-mediated immune modulation, without the safety and ethical concerns associated with conventional immune and/or stem-cell-based approaches.

## Data Availability Statement

The datasets generated for this study are available on request to the corresponding author.

## Author Contributions

YZ supervised experiments and contributed to concepts, experimental design, data analysis, and interpretation, manuscript writing, and final approval of manuscript. WH performed most experiments and data analysis. XS, HY, and JS performed stem cell culture, flow cytometry, and TEM for exosomes.

### Conflict of Interest

YZ is a founder of Tianhe Stem Cell Biotechnology Inc. He is an inventor of Stem Cell Educator technology. The remaining authors declare that the research was conducted in the absence of any commercial or financial relationships that could be construed as a potential conflict of interest.
