# Supplementary figures and images for: Released Exosomes Contribute to the Immune Modulation of Cord Blood-Derived Stem Cells
Source: Front Immunol. 2020 Feb 25;11:165. doi: 10.3389/fimmu.2020.00165 (PMC7052489; doi:10.3389/fimmu.2020.00165)

## Supplementary Data:

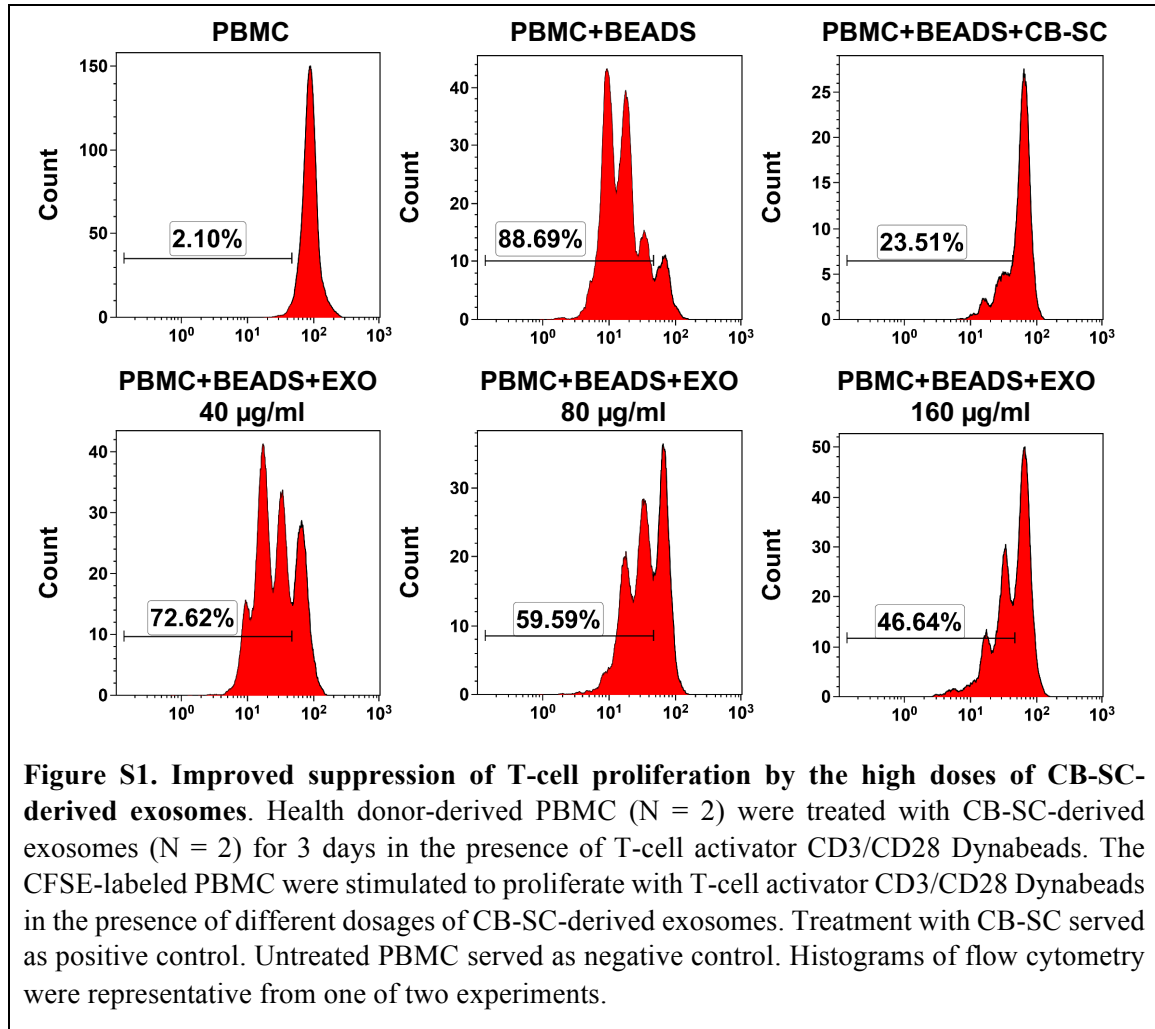

Supplement: Supplementary file 1 [file Data_Sheet_1.pdf]
